# Supplementary material for: Comparison of Pulmonary Function and Inflammation in Children/Adolescents with New-Onset Asthma with Different Adiposity Statuses
Source: Nutrients. 2022 Jul 20;14(14):2968. doi: 10.3390/nu14142968 (PMC9319926; doi:10.3390/nu14142968)
Supplement: Supplementary file 1 [file nutrients-14-02968-s001.zip › nutrients-1779980-supplementary.pdf]

Table S1. Socio-demographic, clinical and immunologic features between normal group and the combined overweight/obese group

	Total	Normal weight	Overweight/Obese	*p*
**Family history of atopy**				
No n(%)	218 (53.6)	111(56.3)	107(51.0)	0.28
Yes n(%)	189(46.4)	86(43.7)	103(49.0)	
**Other allergic disease**				
No n(%)	38(9.3)	22(11.2)	16(7.6)	0.22
Yes n(%)	369(90.7)	175(88.8)	194(92.4)	
Single allergic disease	155(38.1)	74(37.6)	81(38.6)	
Multiple allergic diseases*	214(52.6)	101(51.3)	113(53.8)	
**Positive sIgE test**				
No n(%)	96 (23.6)	53(26.9)	43(20.5)	0.13
Yes n(%)	311(76.4)	144(73.1)	167(79.5)	
**Total IgE (IU/mL)**				
Median	295.00	320.00	281.50	0.17
IQR	135.00-647.00	166.50-649.00	104.50-624.50	
**Eosinophils (*10^9 /L)**				
Median	0.35	0.36	0.35	0.95
IQR	0.13-0.64	0.09-0.68	0.15-0.58	
**Eosinphils%≥4% n(%)**	205(50.4)	95(48.2)	110(52.4)	0.40
**FeNO≥20ppb n(%)**	213(52.3)	107(54.3)	106(50.5)	0.44
**IL-4 (pg/mL)**				
Median	100.05	102.64	98.18	0.38
IQR	88.95-100.59	90.52-110.59	87.36-110.86	

Table S2. Comparison of leptin, adiponectin, serum cytokines levels in different groups

	Total<br>Median (IQR)	Normal weight<br>Median (IQR)	Overweight/ Obese<br>Median (IQR)	*p*
Leptin (ng/mL)	3.18(1.16-6.54)	1.05(0.72-2.70)	4.93(2.24-7.46)	**0.02**
Adiponectin (μg /mL)	68.39(53.00-96.95)	86.42(65.40-126.82)	66.25(45.13-87.21)	**<0.001**#
TNF-α (pg/mL)	30.07(26.45-36.06)	28.75(26.84-31.38)	31.29(25.09-3.26)	0.38
IFN-γ (pg/mL)	7.07(3.98-9.03)	6.54(3.76-8.62)	7.54(4.28-9.11)	0.39
IL-16 (pg/mL)	316.20(233.67-445.66)	237.04(211.00-319.45)	324.30(280.27-485.19)	**0.02**

Bolding indicates $p<0.05$

# indicates if below Bonferroni-adjusted p <0.01(0.05/5outcomes)
